# SLC4A4 promotes prostate cancer progression in vivo and in vitro via AKT-mediated signalling pathway

**DOI:** 10.1186/s12935-022-02546-6

**Published:** 2022-03-19

**Authors:** Zelin Liu, Qinghua Wang, Guanzhong Zhai, Shuai Ke, Xi Yu, Jia Guo

**Affiliations:** Department of Urology, Renmin Hospital of Wuhan University, 9 Zhangzhidong Road, Wuhan, 430060 People’s Republic of China

**Keywords:** SLC4A4 (NBCe1), Prostate cancer, Clinical-stage, Mobility, Mechanisms, SC79

## Abstract

**Background:**

Prostate cancer (PCa) is the second leading cause of cancer-related male deaths worldwide. The purpose of this study was to investigate the effects of homo sapiens solute carrier family 4 member 4 (SLC4A4), which encodes the electrogenic Na^+^/HCO_3_^−^ cotransporter isoform 1 (NBCe1), in the development and progression of PCa.

**Methods:**

The expression levels of SLC4A4 in PCa and normal prostate tissues were evaluated by immunohistochemistry. The SLC4A4 knockdown cell model was structured by lentiviral infection, and the knockdown efficiency was validated by RT-qPCR and Western blotting. The effects of SLC4A4 knockdown on cell proliferation, apoptosis and cycle, migration, and invasion were detected by Celigo cell counting assay and CCK-8 assay, flow cytometry analysis, wound-healing, and Transwell assay, respectively. Tumor growth in nude mice was surveyed by in vivo imaging and Ki-67 staining. Furthermore, underlying mechanism of SLC4A4 silence induced inhibition of PCa progression was explored by human phospho-kinase array.

**Results:**

Our results revealed that SLC4A4 expression was up-regulated in PCa tissues and human PCa cell lines. High expression of SLC4A4 in tumor specimens was significantly correlated with disease progression. SLC4A4 knockdown inhibited cell proliferation, migration and invasion, while facilitated apoptosis, which was also confirmed in vivo. Moreover, SLC4A4 promoted PCa progression through the AKT-mediated signalling pathway.

**Conclusion:**

The results of this study indicated that SLC4A4 overexpression was closely associated with the progression of PCa; SLC4A4 knockdown suppressed PCa development in vitro and in vivo. SLC4A4 acts as a tumor promotor in PCa by regulating key components of the AKT pathway and may therefore act as a potential therapeutic target for PCa treatment.

## Background

Prostate cancer (PCa) is one of the most common cancers in male genitourinary system worldwide and still ranks the second leading cause of cancer-related deaths among men in Western countries [1]. Based on the estimation of GLOBOCAN in 2019, 1,276,106 new cases and 358,989 deaths (3.8% of all male deaths caused by cancer) related to PCa were reported globally [2]. PCa is driven by androgens. Therefore, androgen deprivation therapy in the first-line treatment of locally advanced, biochemically recurrent PCa and metastatic PCa is very valid. Nevertheless, almost all patients who are initially sensitive to androgen deprivation therapy will advance to castration resistance, frequently with metastasis [3]. Although mortality of PCa has been reduced by about 50% as a result of improvements in early detection and treatment [4], the effects of these primary treatments are still limited because the genetic mechanism underlying the occurrence and progression of PCa remains poorly understood. The cellular carcinogenesis process is multi-step and complex, involving multiple factors and genes [5, 6], along with alterations in the expression modes of various genes, which in turn influence cell proliferation, apoptosis and differentiation [7]. PCa is a highly heritable disease with a strong genetic component. Thus, it is particularly significant to identify the genetic risk factors for PCa and search for new therapeutic targets.

Homo sapiens solute carrier family 4 member 4 (SLC4A4) is a family member of the solute carrier family and encodes an electrogenic Na^+^/HCO_3_^−^ cotransporter, which is mainly involved in the secretion and absorption of sodium bicarbonate [8]. This process is highly important for maintaining the dynamic pH equilibrium within cells. SLC4A4 and other solute carrier family members have been found to be associated with tumorigenesis and tumor development [9]. For instance, Destruction of SLC4A4 or SLC4A9 by genetic or pharmacological methods have been reported to acidify intracellular pH and suppress cancer cell growth [10]. Accumulating evidence showed that the expression of SLC4A4 is different in a variety of malignant tumors. MicroRNA 223-3p inhibited the expression of SLC4A4 in clear cell renal cell carcinoma, promoting the cancer proliferation and metastasis [11, 12]. SLC4A4 expression was also shown to be down-regulated in thyroid cancer, providing diagnostic efficacy in clinical practice [13]. Moreover, SLC4A4 expression was shown to be higher in chronic myeloid leukemia and mucinous epithelial ovarian cancer than in adjacent normal tissue [14], suggesting that the biological processes in which SLC4A4 is involved are tumor-specific. Although SLC4A4 can indicate the prognosis of patients with colon adenocarcinoma and some other kinds of tumors [15, 16], its significance in PCa has not been revealed.

## Materials and methods

### Patient specimens and immunohistochemical staining

Tumour tissues and adjacent paired non-tumour tissues were gathered from patients who were diagnosed with PCa and underwent surgical excision in Renmin Hospital of Wuhan University (Wuhan, China) between June 2018 and January 2020. PCa and normal prostate tissues were gathered from 74 patients; the age range of the patients was between 26 and 87(mean age, 65 years).

Immunohistochemistry (IHC) was used to detect the expression of SLC4A4 in these tissues. Paraffin-embedded sections were dewaxed, the antigen was retrieved, and then the paraffin sections were incubated using primary antibody against SLC4A4 (cat. No. bs-21660R; Bioss) (1:200) and secondary antibody goat anti-rabbit (cat. No. A0208; Beyotime) (1:400). After staining, ten fields (× 100 magnification) were chosen to be captured and analyzed with the optical microscope (Olympus, Japan) for each section. The SLC4A4 staining intensity was scored on a range from 0 (negative), 1 (weak), 2 (positive + +) and 3 (positive +  + +). Median IHC score was used to distinguish the high/low expression of SLC4A4.

### Cell culture

Human PCa cell lines DU 145, LNCaP and PC-3 were purchased from the Cell Bank of Chinese Academy of Sciences and cultured in RPMI-1640 medium at 37 °C in a humidified incubator containing 5% CO_2_. The cell culture media were supplemented with 10% fetal bovine serum (FBS) and 1% sodium penicillin G/streptomycin sulfate (P/S). The normal prostate epithelial cell line RWPE-1 was purchased from the Cell Bank of Chinese Academy of Sciences and cultivated in keratinocyte serum-free medium (K-SFM) containing 0.05 mg/mL bovine pituitary extract (BPE), 5 ng/mL epidermal growth factor (EGF) and 1% P/S.

### Construction of target gene interference lentivirus

To knock down the expression of SLC4A4 in PCa cell lines, the short hairpin RNA (shRNA) sequence targeting the human *SLC4A4* gene was identified as 5'- TTATTCTTCAGCTGGTCCTTC-3'. Meanwhile, the target sequence of the negative control shRNA was identified as 5'-TTCTCCGAACGTGTCACGT-3'. Oligomers were annealed and ligated to BR-V121 lentiviral vector (Yibeirui, Shanghai, China) through Age I/EcoR I restriction site to produce Lv-shSLC4A4 and Lv-shCtrl. Lastly, the sequencing was performed to validate the construct results.

The modified BR-shRNA plasmid and pMD2.G and pSPAX2 helper plasmids were transfected three times with Lipofectamine 2000 into HEK-293 T cells to obtain lentiviruses. Next, the lentiviral particles were gathered, filtered, and preserved. The knockdown efficiency of SLC4A4 was evaluated by RT-qPCR and Western blotting.

### Cell transfection and fluorescence imaging

PCa cells were respectively transfected with 1 × 10^7^ TU/ml of the lentivirus containing shRNA interfering with SLC4A4 (shSLC4A4) or shRNA for negative control (shCtrl), and the cells were then incubated at 37 °C for three days. The expression of green fluorescent protein (GFP, carried by the lentiviral vector) was observed by fluorescent microscope (EMD Millipore), and the ratio of fluorescent cells to total cells (viewed in white light) was used to evaluate the transfection efficiency.

### Reverse transcription and real-time quantitative PCR (RT-qPCR) assays

PCa cells were gathered and centrifuged. Total RNA was extracted with the TRIzol reagent. Complementary DNAs (cDNAs) was synthesized with the PrimeScript™ RT reagent Kit (Takara). The qPCR reaction system was prepared using a real-time quantitative PCR instrument according to product specification. The reaction system was composed of the following reagents: TB Green® *Premix Ex Taq*™ II, Forward and Reverse primers (Sangon Biotech), reverse transcription products and RNase-free H_2_O. The thermal cycling conditions were: pre-denaturation at 95 °C for 30 s, denaturation at 95 °C for 15 s, annealing at 60 °C for 10 s for a total of 42 cycles, and 72 °C for 5 min (extension). GAPDH was used as an internal reference. The relative expression levels of genes were calculated as the method of the 2^−ΔΔCt^ [17]. The sequences of the main primers are as follows: GAPDH forward, 5'-TGACTTCAACAGCGACACCCA-3' and reverse, 5'-CACCCTGTTGCTGTAGCCAAA-3'; SLC4A4 forward, 5'-AAGCTCTTTCGGCAATTCTCTTC-3' and reverse, 5'-GAAACTCTCCAACACGCCCTG-3'.

### Western blotting

PCa cells were washed twice with ice-cold PBS, lysed with cell lysis buffer (Beyotime) containing protease inhibitors, and incubated on ice for 15 min. The supernatant was harvested by centrifugation, and the protein content was measured with the BCA Protein Assay Kit (cat. No. 23225; HyClone-Pierce). Equal amounts of proteins were separated and transferred onto polyvinylidene difluoride (PVDF) membranes (0.45 μM). Subsequently, the membranes were blocked with TBS + 0.1% Tween-20 (TBST) buffer containing 5% skim milk and incubated with various primary antibodies. Following being washed with TBST, the membranes were blotted with HRP-conjugated secondary antibodies. The target bands were visualized with immobilon Western Chemiluminescent HRP Substrote (cat. No. RPN2232; Millipore). GAPDH was used as an internal reference.

The antibodies used in this experimental study were BAX (cat. No. 50599–2-Ig; Proteintech), BCL-2 (cat. No. sc-7382; santa cruz), CDK4 (cat. No. 11026–1-AP; Proteintech), FAS (cat. No. 13098–1-AP; Proteintech), AKT (cat. No. 10176–2-AP; Proteintech), p-AKT (cat. No. 66444–1-Ig; Proteintech), GAPDH (cat. No. 60004–1-lg; Proteintech), horseradish peroxidase (HRP)-conjugated goat anti-rabbit IgG (cat. No. A0208; Beyotime), goat anti-mouse (cat. No. A0216; Beyotime), donkey anti-goat (cat. No. A0181; Beyotime).

### Celigo cell counting assay

Following infection with shRNA lentivirus, PCa cells were trypsinized, resuspended, counted and then inoculated in 96-well plates. Each group underwent a minimum of 3 duplicate wells. From the second day, the plates were tested by Celigo Imaging Cytometry System once a day for 5 consecutive days. The number of green fluorescent cells in each scan plate was precisely calculated by adjusting the input parameters of the analysis setup; the data were then plotted statistically, and the cell proliferation curve for 5 days was drawn.

### Cell Counting Kit-8 (CCK-8) assay

CCK-8 (Dojindo, Shanghai) assay was used to determine the number of living cells. The cell suspension was inoculated with 2 × 10^3^ cells per well into a 96-well plate and pre-incubated for 24 h after transfection. Treated or un-treated cells were cultured as appropriate. 10 µl of CCK-8 solution was added to each well, and the cells were incubated for 2 h at 37 °C. The absorbance at 450 nm was recorded with a TECAN infinite M200 Multimode microplate reader (Tecan, Mechelen, Belgium). All detections were conducted in triplicate.

### Flow cytometry analysis

Apoptosis was detected by the flow cytometry analysis. The infected PCa cells were cultured until the cell density reached 85%. The cells were trypsinized and resuspended, centrifuged for 5 min. The supernatant was discarded, and cell precipitates were washed with D‑Hank's solution (pH = 7.2–7.4) precooled at 4 °C. The cells were washed with 1 × binding buffer, centrifuged, and resuspended. The cell suspensions (1 × 10^5^–1 × 10^6^ cells) were stained by 10 µl Annexin V-APC and protected from light for 15 min [18]. Subsequently, 400–800 µl of 1 × binding buffer was added depending on the amount of cells. At last, the cells were tested by Guava easyCyte HT flow cytometer and analysed with FlowJo VX10.

Cell cycle distribution was analysed with the flow cytometry. When 6 cm dish cells in each experimental group grew to about 80% coverage, the cells were fixed at least 1 h after the washing. Afterwards, cells were washed and resuspended in PBS containing PI and RNase A. Finally, the cells were tested by flow cytometer, and the percentage of the cells in G0/G1, S and G2/M phases were visualized by a ModFit. All detections were performed in triplicate.

### Scratch test

The aim of this assay was to assess the migration ability of cells after transfection. When the cells were dense in the microscopic field, three standardized wounds per well were scratched with the tip of a sterile pipette. Then, the cells were cultured in serum-free medium. Wound sizes were photographed with a phase-contrast microscope at 0 and 24 h, respectively. The five randomly selected fields were adopted to calculate the rate of wound healing using ImageJ software.

### Transwell invasion assay

Diluted Matrigel (Corning, USA) was added to transwell upper chambers 12 h prior to the experiment and placed at 37 °C for solidification. The PCa cells (1.0 × 10^5^ cells per chamber) were inoculated in the upper chambers by resuspension with basal serum-free medium, and the lower chambers was added with medium containing 20% FBS to attract cells to penetrate the membrane. Following incubation for 24 h, those cells on the outside surface of the chambers, having invaded through the membrane, were fixed using 4% paraformaldehyde (500 µl per chamber) for 20 min and stained using 0.1% crystal violet for 20 min. Cells on the upper surface and remaining Matrigel were wiped off by cotton swabs. At last, the numbers of stained cells in five different views per chamber were counted.

### Xenograft animal model

To study in vivo tumor growth, four-week-old BALB/c nude mice, weighing 18–20 g, were purchased from Beijing HFK Bioscience Co. Ltd. All 20 mice were placed in SPF housing conditions.

After a week of acclimatization in Animal Experiment Center of Renmin Hospital of Wuhan University, 20 mice were randomly divided into the control group (shCtrl) and the test group (shSLC4A4) (n = 10 mice/group). Since LNCaP was an androgen-dependent cell line, the tumorigenic rate of subcutaneous inoculation in nude mice alone was very poor [19]. DU 145, on the other hand, was an androgen-independent line with low differentiation and better tumorigenic effect [20, 21]. The shRNA lentivirus-infected DU 145 cells were digested, suspended and injected subcutaneously into right forelimb axilla of each mouse (serum-free medium containing 4 × 10^6^ cells). Those 20 mice were reared for 31 days, during which length and width of the tumors in mice were measured five times using the Vernier caliper. On day 31, the mice were injected intraperitoneally with D-Luciferin, anesthetized with an intraperitoneal injection of 0.7% pentobarbital sodium and placed under the animal multispectral living imaging system for imaging. Next, the mice were sacrificed with cervical dislocation, and tumors autopsied from the mice were weighed and photographed. Tumor volume in mm^3^ (V) was calculated based on the formula: V = π/6 × L × W × W, where L represents length and W represents width of the tumor.

### Ki-67 staining

Paraffin sections of tumor tissues taken from mice were dewaxed, rehydrated in a decreasing ethanol gradient, and incubated with the anti-Ki-67 antibody (cat. No. ab16667; Abcam). After washing with PBS, the paraffin sections were incubated with secondary antibody goat anti-rabbit (cat. No. ab97080; Abcam), counterstained with hematoxylin, and Ki‑67 expression was observed under an optical microscope. Ten fields of each section were captured for analysis, and this experiment was repeated three times.

### Human Phospho-Kinase array (proteome profiler)

To explore the potential downstream signal pathways and functional targets of SLC4A4 in PCa, Human Phospho-Kinase Array Kit (cat. no. ARY003C; Bio-Techne China Co., Ltd.) was employed. PCa cells transfected with shCtrl or shSLC4A4 were lysed. Meanwhile, 8 nitrocellulose membranes (4 Part A, 4 Part B, each containing 39 different capture antibodies printed in duplicate) were closed with 2 ml of Array Buffer 1 (block buffer) for 1 h. Then, the samples were piped to wells and incubated overnight. After washing, 1 × biotinylated antibody cocktail was added to each well and incubated. Afterwards, diluted Streptavidin-HRP was pipetted into each well and incubated. Followed by the washing of membranes, any excess Wash Buffer was blotted off, and 500 µl of Chemi Reagent Mix (equal vol. of Chemi Reagent 1 and 2) was added to each membrane. In the end, the signal density was measured with the chemiluminescence imaging system and analysed with the ImageJ. This experiment was performed in duplicate.

### Statistical analysis

SPSS 23.0 and Graphpad Prism 8 were used for data analysis. The quantitative data were presented as the mean ± standard deviation (SD). Chi-squared tests were performed to compare the differences in SLC4A4 expression among PCa patients. Spearman rank correlation analysis was used to analyse the correlation between SLC4A4 expression and clinicopathological features. The histograms of SLC4A4-related signal molecules in carcinoma cell were plotted by SignaLink 2.0 analysis. P-value < 0.05 was considered a statistically significant difference.

## Results

### Expression of SLC4A4 in clinical prostate specimens

**Fig. 1:**
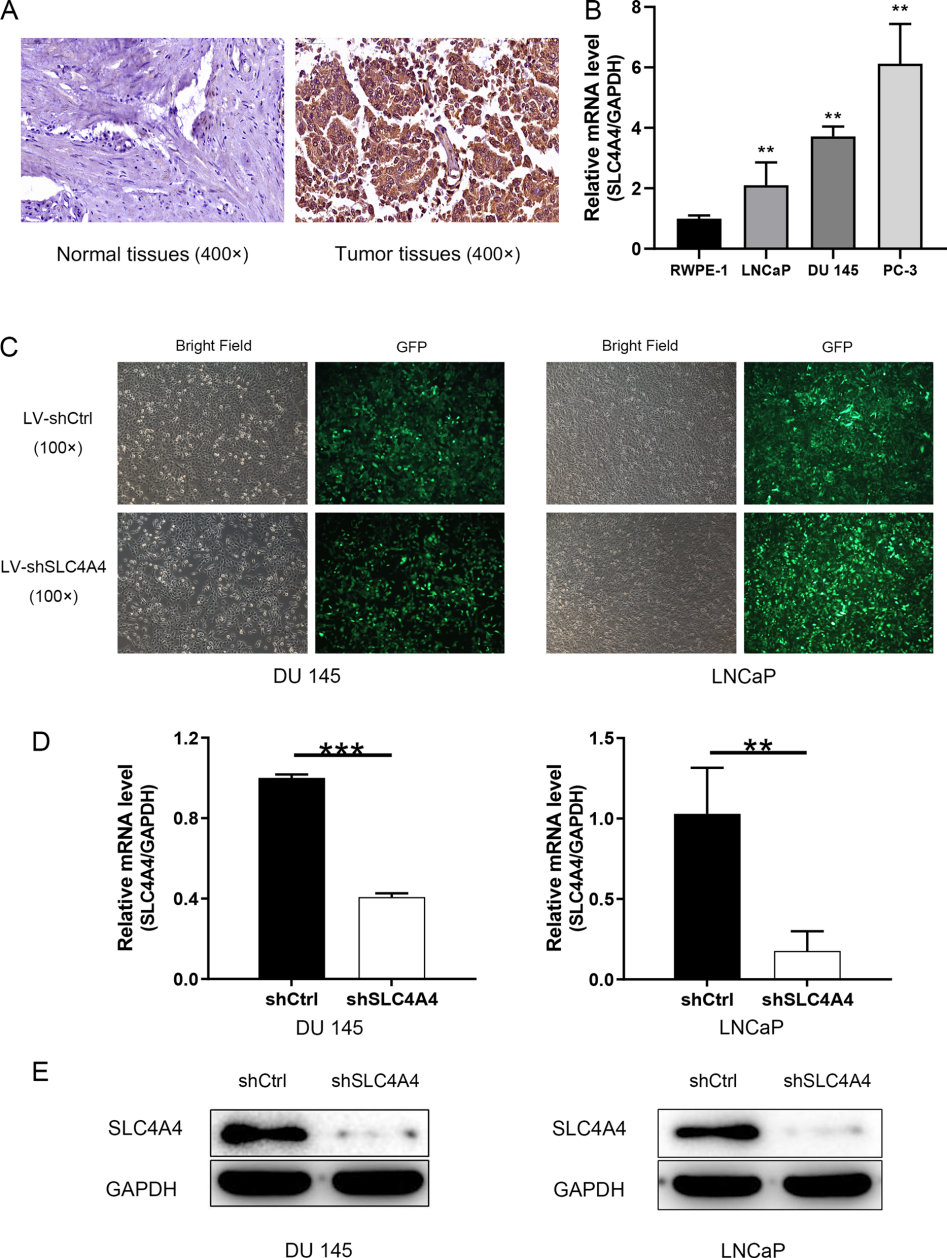
Expression of SLC4A4 in prostate specimens and cell lines and construction of the SLC4A4-knockdown cell model. **A** Representative images of IHC: Low expression of SLC4A4 in normal prostate tissues and high expression of SLC4A4 in PCa tissues (magnification, × 400). **B** SLC4A4 mRNA was significantly up-regulated in PCa cell lines (LNCaP, DU 145 and PC-3) compared with the normal prostate epithelial cell line (RWPE-1). **C** Transfection efficiency of DU 145 and LNCaP cells was checked using fluorescence imaging (magnification, × 100). **D** SLC4A4 mRNA expression was detected by RT-qPCR in each group of DU 145 and LNCaP cells. **E** The expression levels of SLC4A4 protein were determined by western blotting in DU 145 and LNCaP cells. Error bars indicate the mean ± SD of at least three replicate experiments. ***P < 0.001, **P < 0.01. SLC4A4, solute carrier family 4 member 4; shSLC4A4, cells transfected with the lentivirus containing shRNA targeting SLC4A4; shCtrl, cells transfected with the lentivirus containing shRNA of negative control

For the purpose of determining the effect of SLC4A4 on the development of PCa, the expression of SLC4A4 in clinical PCa and normal prostate tissues was examined by IHC. As demonstrated in Fig. 1A, the results of 192 pathological sections confirmed the cytoplasmic localization of SLC4A4 and indicated that the expression of SLC4A4 in PCa tissues was distinctly higher than that in normal prostate tissues (P < 0.001; Table 1), which can be used for follow-up statistical analysis of clinicopathological data.

**Table 1:** Expression patterns of SLC4A4 in clinical prostate tissues revealed in immunohistochemistry analysis

SLC4A4 expression	Tumor tissue		Normal prostate tissue		p value
	Cases	Percentage	Cases	Percentage	
Low	29	51.8%	17	94.4%	< 0.001
High	27	48.2%	1	5.6%	

### Expression of SLC4A4 in clinicopathological data of PCa patients

The relationship between the levels of SLC4A4 expression and the clinicopathological characteristics in PCa patients was assessed by statistical analysis. The high/low SLC4A4 groups were divided depending on median of IHC scores of all tissue specimens. The results demonstrated that the levels of SLC4A4 expression were remarkably different between patients at different clinical stages, T Infiltrate and lymphatic metastasis (P < 0.05; Table 2). According to Spearman rank correlation analysis, the clinical stage, T Infiltrate and the risk of lymphatic metastasis were positively correlated with high SLC4A4 expression (Table 3). These results suggested that the expression of SLC4A4 was increasing with the deepening of tumour malignancy.

**Table 2:** Relationship between SLC4A4 expression levels and clinicopathological features in patients with prostate cancer

Clinical parameters	No. of patients	SLC4A4 expression		p-value
		Low	High	
All patients	56	29	27	
Age (years)				0.502
< 69	27	15	12	
≥ 69	28	13	15	
Gleason score				0.168
< 7	18	11	7	
≥ 7	32	13	19	
Grade				0.632
1	8	5	3	
2	25	11	14	
3	17	8	9	
T Infiltrate				< 0.001
T2	37	26	11	
T3	15	2	13	
T4	4	1	3	
Lymphatic metastasis (N)				0.016
N0	51	29	22	
N1	5	0	5	
Metastasis				0.139
M0	54	29	25	
M1	2	0	2	
Stage				< 0.001
I	14	11	3	
II	22	15	7	
III	13	2	11	
IV	7	1	6	
Gleason Grade				0.516
2	5	2	3	
3	13	9	4	
4	15	5	10	
5	17	8	9

**Table 3:** Correlation between SLC4A4 expression and tumor characteristics in patients with prostate cancer

		SLC4A4
T Infiltrate	Spearman correlation	0.500
	Significant (double tail)	< 0.001
	N	56
Lymphatic metastasis (N)	Spearman correlation	0.325
	Significant (double tail)	0.015
	N	56
Stage	Spearman correlation	0.516
	Significant (double tail)	< 0.001
	N	56

### Knockdown of SLC4A4 in PCa cells

The results of qRT-PCR verified that SLC4A4 mRNA expression was significantly higher in the PCa cell lines than in the normal prostate epithelial cell line RWPE-1 (Fig. 1B). For investigating the roles of SLC4A4 in PCa, SLC4A4-targeting shRNA was cloned into GFP-carrying lentiviral vector. Afterwards, shSLC4A4 or shCtrl lentivirus was transfected into human PCa cell lines. Where the detailed plots of DU 145 and LNCaP are presented in Fig. 1C. The fluorescent signal inside cells, which have been infected with shCtrl or shSLC4A4 for 72 h, observed by the microscope, reveal a > 80% transfection efficiency in both cell lines. The knockdown efficiency of SLC4A4 was examined using RT-qPCR on mRNA level. The results demonstrated that, compared with shCtrl group, the knockdown efficiency of SLC4A4 in shSLC4A4 group was 59.24% (P < 0.001) in DU 145 cells; the knockdown efficiency of SLC4A4 in shSLC4A4 group was 82.79% in LNCaP cells (P < 0.01; Fig. 1D). Furthermore, results of western blotting also indicated that the expression of SLC4A4 protein was distinctly down-regulated after infection in comparison with shCtrl cells (Fig. 1E). These results implied that the SLC4A4-knockdown cell models were successfully constructed.

### Knockdown of SLC4A4 inhibits PCa cell proliferation and facilitates apoptosis

**Fig. 2:**
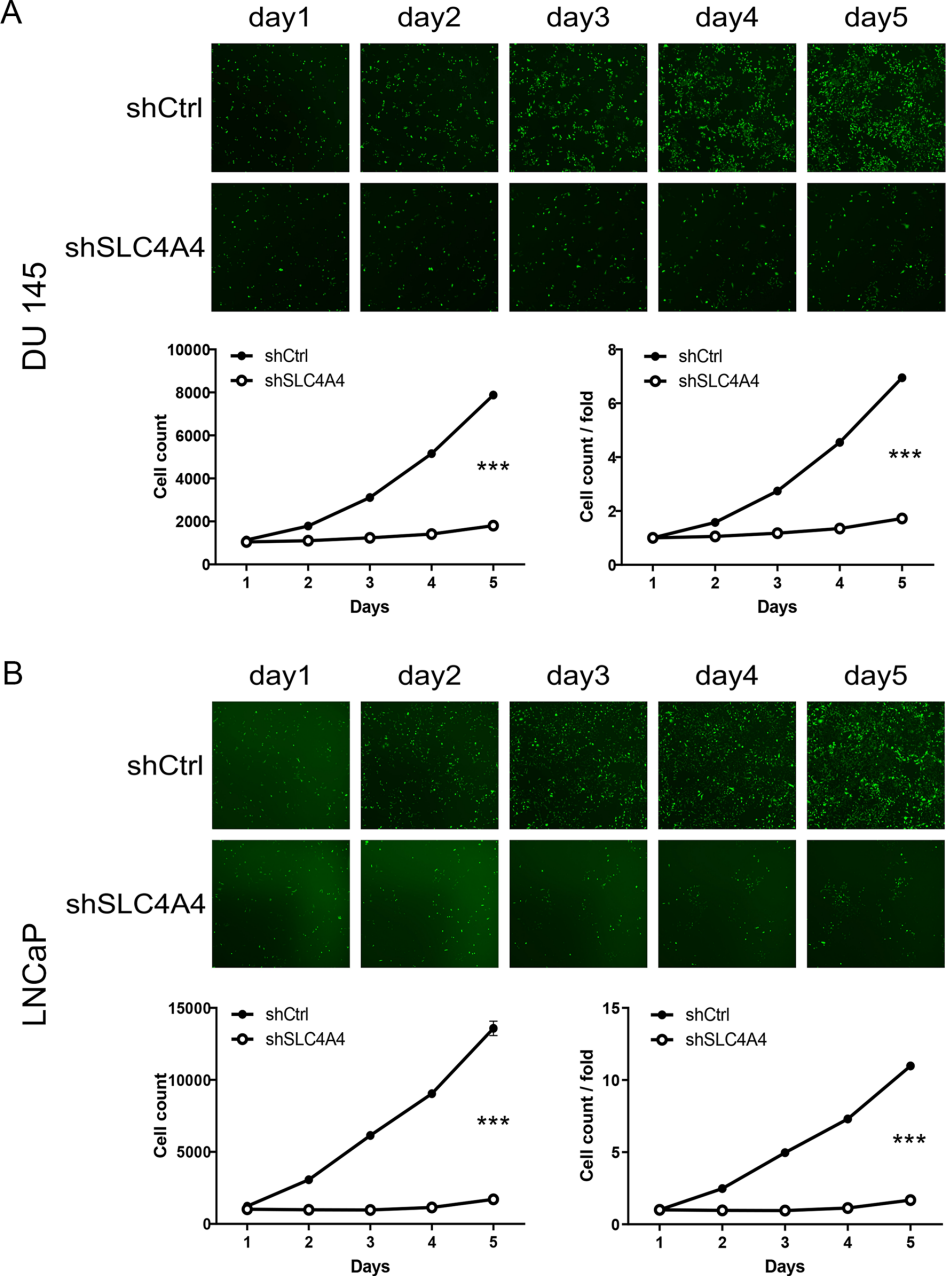
SLC4A4 knockdown restrains the proliferation of DU 145 and LNCaP cells. Effects of SLC4A4 knockdown on the survivability of DU 145 (**A**) and LNCaP (**B**) cells were determined using Celigo cell counting assay. The shSLC4A4 groups showed a dramatic decrease in growth rate in DU 145 and LNCaP cells compared to the shCtrl group. Error bars indicate the mean ± SD of at least three replicate experiments. ***P < 0.001

**Fig. 3:**
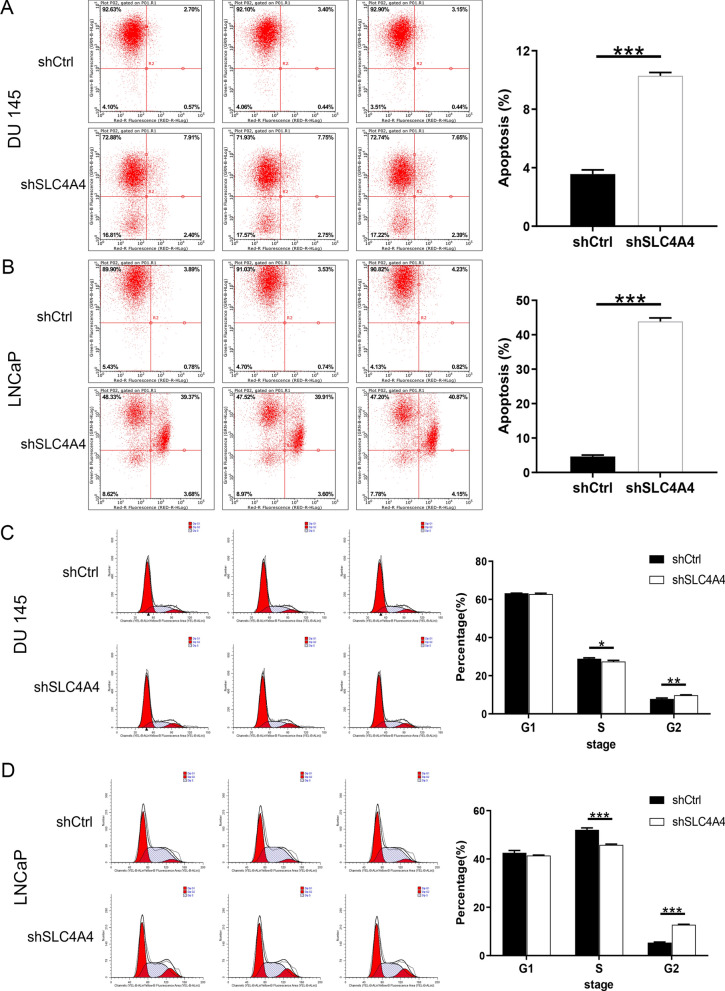
Effects of SLC4A4 on apoptosis and cell cycle of PCa cells. The percentages of apoptosis in each group in DU 145 cells (**A**) and LNCaP cells (**B**) were assayed using flow cytometry, the apoptotic rate of PCa cells was noticeably higher in shSLC4A4 group compared to shCtrl group. **C**, **D** Flow cytometry assay was employed to analyse cell cycle. Error bars indicate the mean ± SD of at least three replicate experiments. ***P < 0.001, **P < 0.01, *P < 0.05

To observe the effect of SLC4A4 on cell proliferation, the growth curves of PCa cells in 5 days were depicted by Celigo Imaging Cytometry System. This is presented in Fig. 2A, B, cell proliferation is obviously suppressed in shSLC4A4 group in comparison with shCtrl group. In both DU 145 and LNCaP cells, the cells of shSLC4A4 group exhibited a slower proliferation rate (P < 0.001, fold change = − 4 and − 6.5, respectively). Flow cytometry analysis was used for detecting the effect of SLC4A4 knockdown on PCa cells apoptosis. Compared with the shCtrl group, the percentage of apoptotic cells in shSLC4A4 group was increased by 2.9-fold in DU 145 cells and 9.4-fold in LNCaP cells (P < 0.001; Fig. 3A, B), implying that SLC4A4 knockdown facilitated apoptosis among PCa cells. Furthermore, it was found that, after SLC4A4 knockdown, more cells were stalled in G2 phase and fewer in G1 and S phases than those transfected with shCtrl (Fig. 3C, D). Collectively, knockdown of SLC4A4 inhibited PCa development in vitro.

### Knockdown of SLC4A4 inhibits the mobility of PCa

**Fig. 4:**
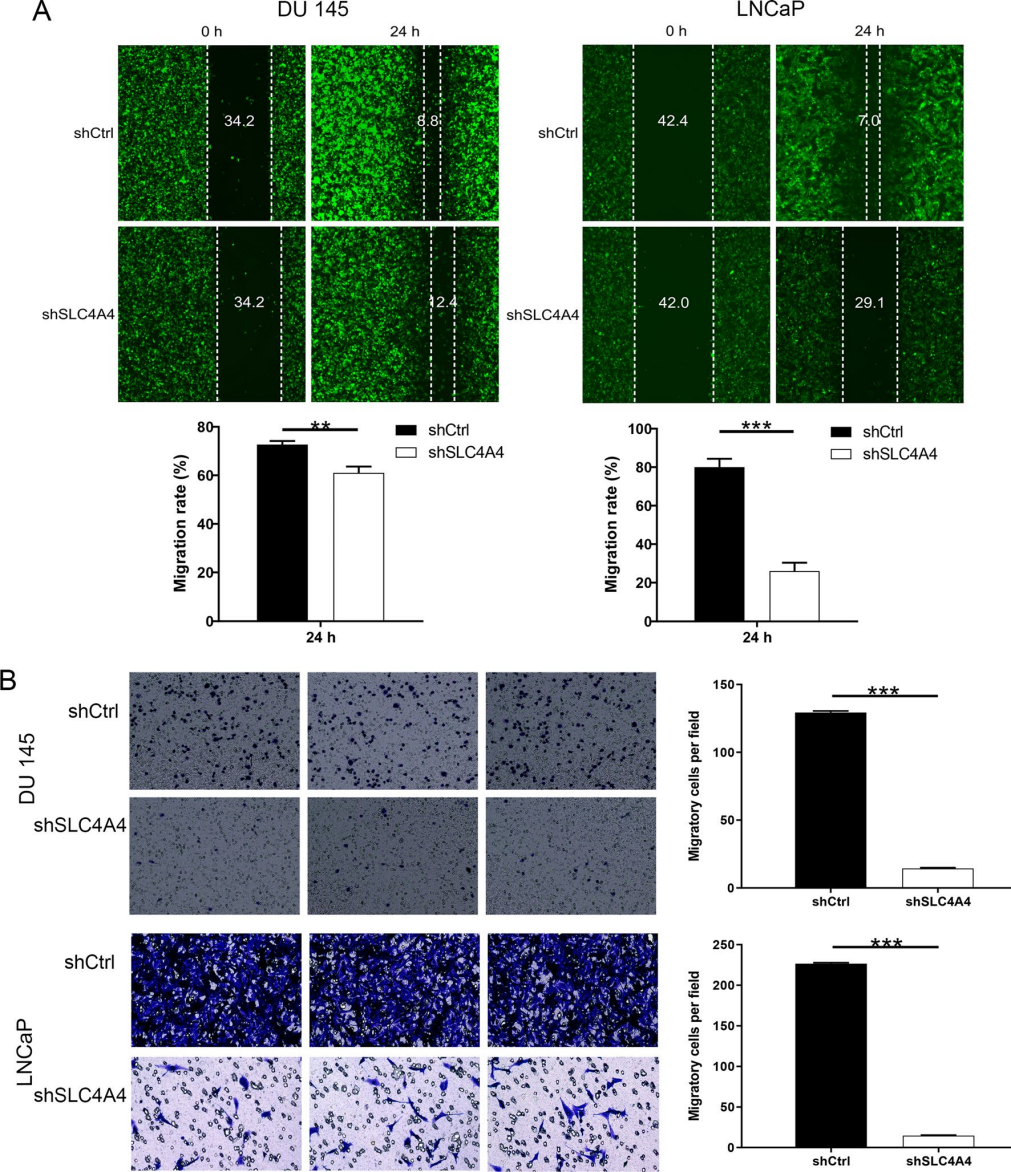
Effects of SLC4A4 on the mobility of PCa cells. **A** The migratory ability of shRNA-infected cells was assayed by scratch test, the wounds were photographed under 100 × magnification. **B **The invasive ability of shRNA-infected cells was examined by transwell assays which were photographed under 200 × magnification. Error bars indicate the mean ± SD of at least three replicate experiments. ***P < 0.001, **P < 0.01

Scratch test detected that, after being transfected with the corresponding lentivirus, in contrast to shCtrl group, the migration rate of cells in shSLC4A4 group (24 h) was reduced by 16% (P < 0.01) in DU 145 cells and 68% (P < 0.001) in LNCaP cells (Fig. 4A), respectively. Simultaneously, the outcomes of transwell assay suggested that, the invasion rate of cells in shSLC4A4 group was reduced by 89% (P < 0.001) in DU 145 cells and 93.6% (P < 0.001) in LNCaP cells (Fig. 4B).

### Effect of SLC4A4 knockdown on tumour progression in vivo

**Fig. 5:**
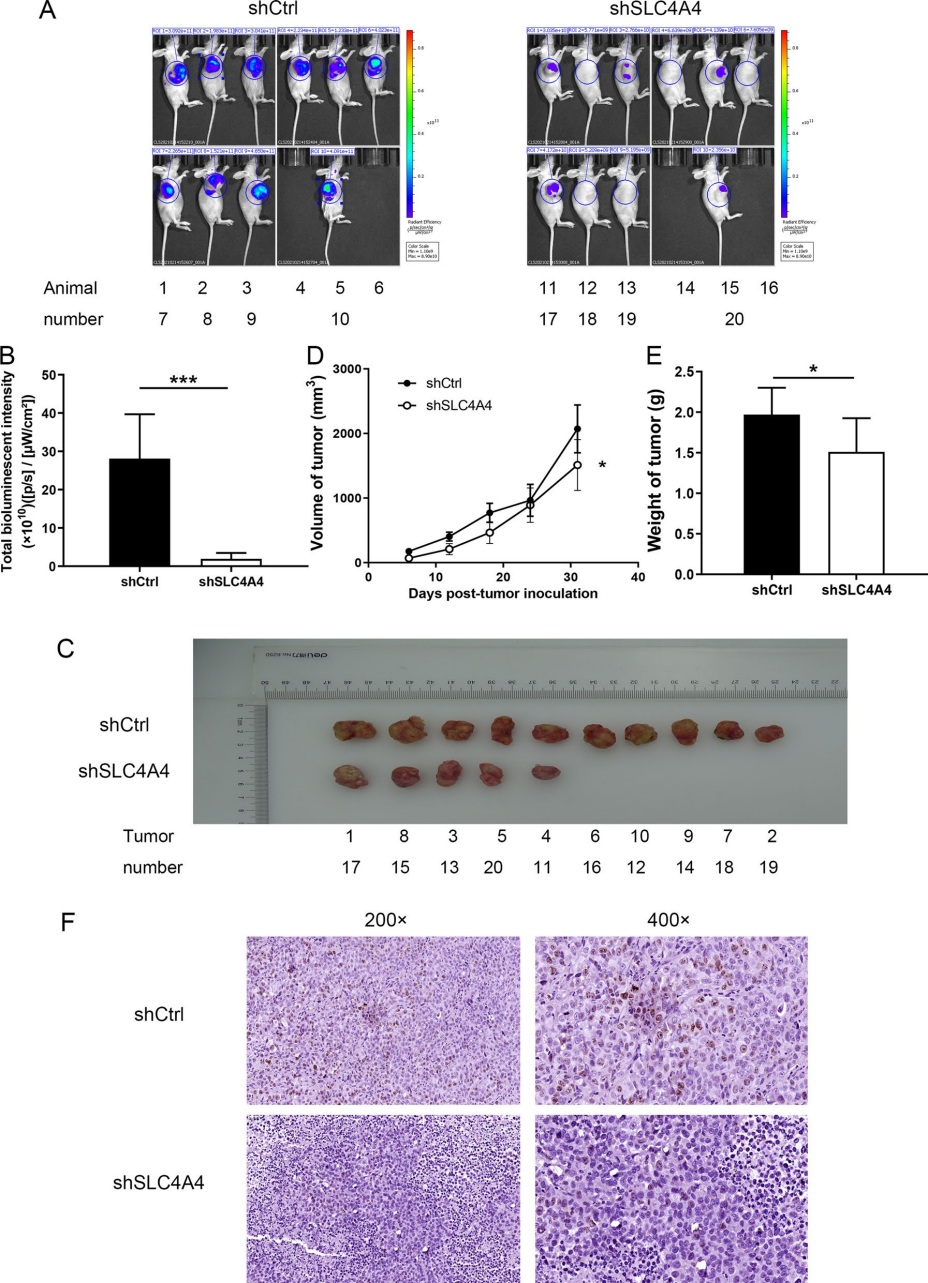
Effect of SLC4A4 knockdown on PCa progression in vivo. **A** In vivo imaging results of xenograft tumors. **B** The bioluminescence intensity of shSLC4A4 group was distinctly diminished compared to shCtrl group. **C** Tumors dissected from the mice. **D** Volume changes of tumors in the two groups during the whole experiment. **E** Mean weight of tumors in each group. **F** Ki-67 expression in tumors resected from mice under 200 × and 400 × magnification. Error bars indicate the mean ± SD of at least three replicate experiments. ***P < 0.001, *P < 0.05. shSLC4A4, mice injected with cells transfected with short hairpin RNA targeting SLC4A4; shCtrl, mice injected with cells transfected with control shRNA

In order to investigate the potential of shSLC4A4 as a therapeutic target for PCa, the nude mouse xenograft model was established with DU 145 cells. The transfection efficiency of infecting into DU 145 cells with shSLC4A4 or shCtrl lentivirus was identified to be > 80%, and these cells were then injected subcutaneously into the 20 mice. After intraperitoneal injection of D-fluorescein into the 20 mice, the bioluminescence intensities (µW/cm^2^) were calculated by in vivo imaging. In contrast to shCtrl group, the bioluminescence intensity of shSLC4A4 group was lowered by 93% (P < 0.001; Fig. 5A, B). In addition, compared to shCtrl group, the tumors from mice of shSLC4A4 group were smaller in diameter at all five measured stages, and were lower in weight (P < 0.05; Fig. 5C–E). The above results indicated that the tumor growth was slower in shSLC4A4 group (P < 0.05). Besides, Ki-67 staining showed that the proliferative potential of PCa cells was obviously restrained in shSLC4A4 group in contrast to shCtrl group (Fig. 5F). These results support that SLC4A4 knockdown can inhibit tumour progression in vivo. Altogether, the findings imply that shSLC4A4, which targets explicitly SLC4A4, may have a potent prohibitive effect on prostate tumorigenesis in vivo.

### Regulatory mechanisms of SLC4A4 in PCa

**Fig. 6:**
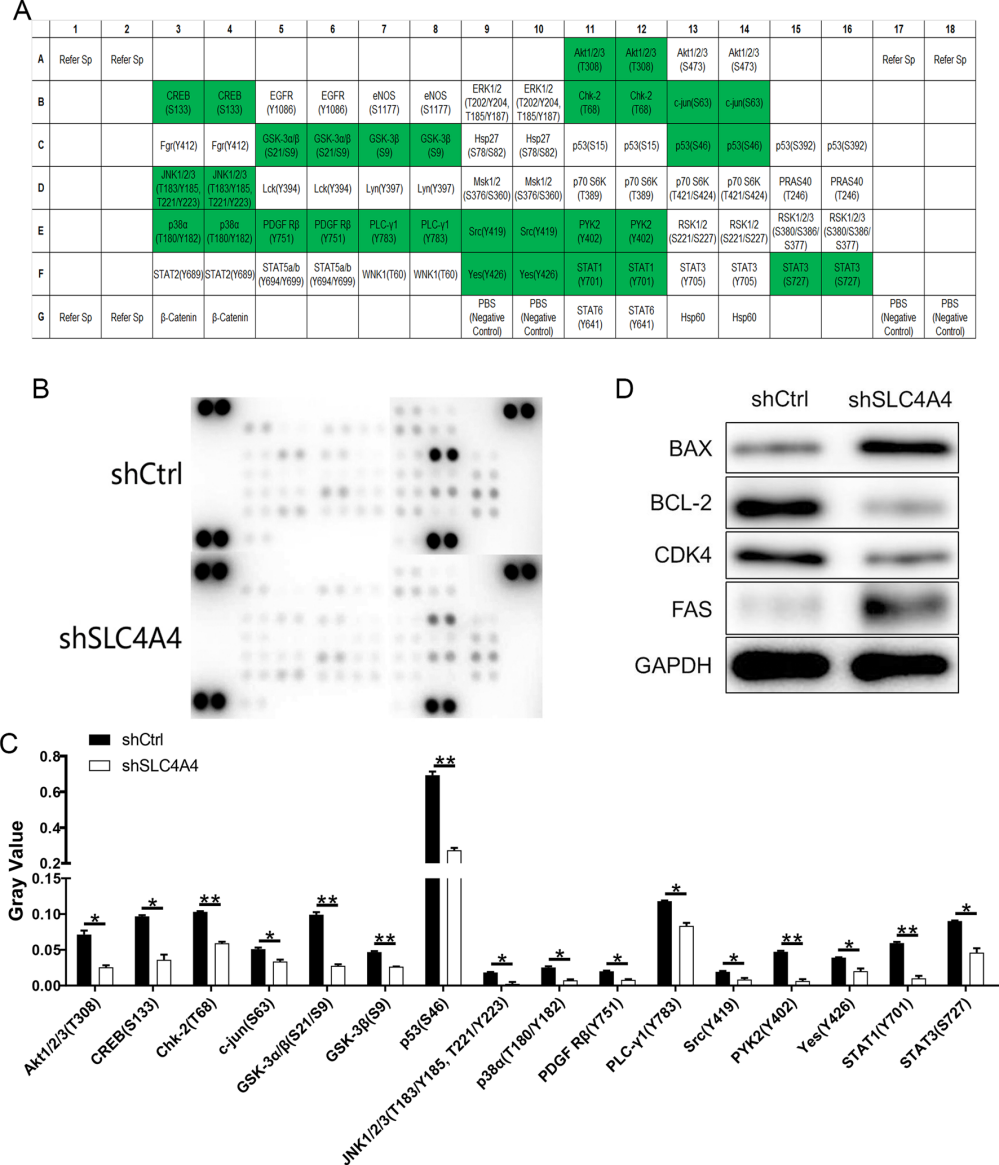
The exploration of regulatory mechanism of PCa by SLC4A4 silencing. **A** Proteins alignment and distribution in the intracellular signalling array. **B** Protein expression findings in the intracellular signalling array with or without SLC4A4 knockdown. **C** Histograms of SLC4A4-related signalling molecules in carcinoma cell analyzed by SignaLink 2.0. **D** Verification of the expression of target proteins. Error bars indicate the mean ± SD of at least three replicate experiments. **P < 0.01, *P < 0.05

The potential downstream signalling pathways and functional targets mediating the effects induced by SLC4A4 knockdown on PCa were explored using Human Phospho-Kinase Array Kit. Among them, the expression levels of proteins Akt1/2/3 (T308), CREB (S133), Chk-2 (T68), c-jun (S63), GSK-3α/β (S21/S9), GSK-3β (S9), p53 (S46), JNK1/2/3 (T183/Y185, T221/Y223), p38α (T180/Y182), PDGF Rβ (Y751), PLC-γ1 (Y783), Src (Y419), PYK2 (Y402), Yes (Y426), STAT1 (Y701) and STAT3 (S727) were assayed to be a significant down-regulation upon SLC4A4 knockdown compared with the shCtrl-infected cells (P < 0.05; Fig. 6A–C), suggesting the potential functional targets for SLC4A4-related regulation of PCa and guiding future directions of our research.

### Variation of expression of related proteins after SLC4A4 knockdown

The results of western blotting indicated that after SLC4A4 interfered, compared with shCtrl group, the expressions of proteins CDK4, BCL-2 were down-regulated. In contrast, the expressions of proteins BAX, FAS were up-regulated (Fig. 6D). These protein bands have further verified that the knockdown of SLC4A4 could induce PCa apoptosis and boost the BAX/BCL-2 ratio, exhibiting a potent effect of SLC4A4 on restraining apoptosis and cell cycle. In other words, the results indicate that SLC4A4 promotes the progression of PCa.

### SLC4A4 promotes PCa progression through the AKT pathway

**Fig. 7:**
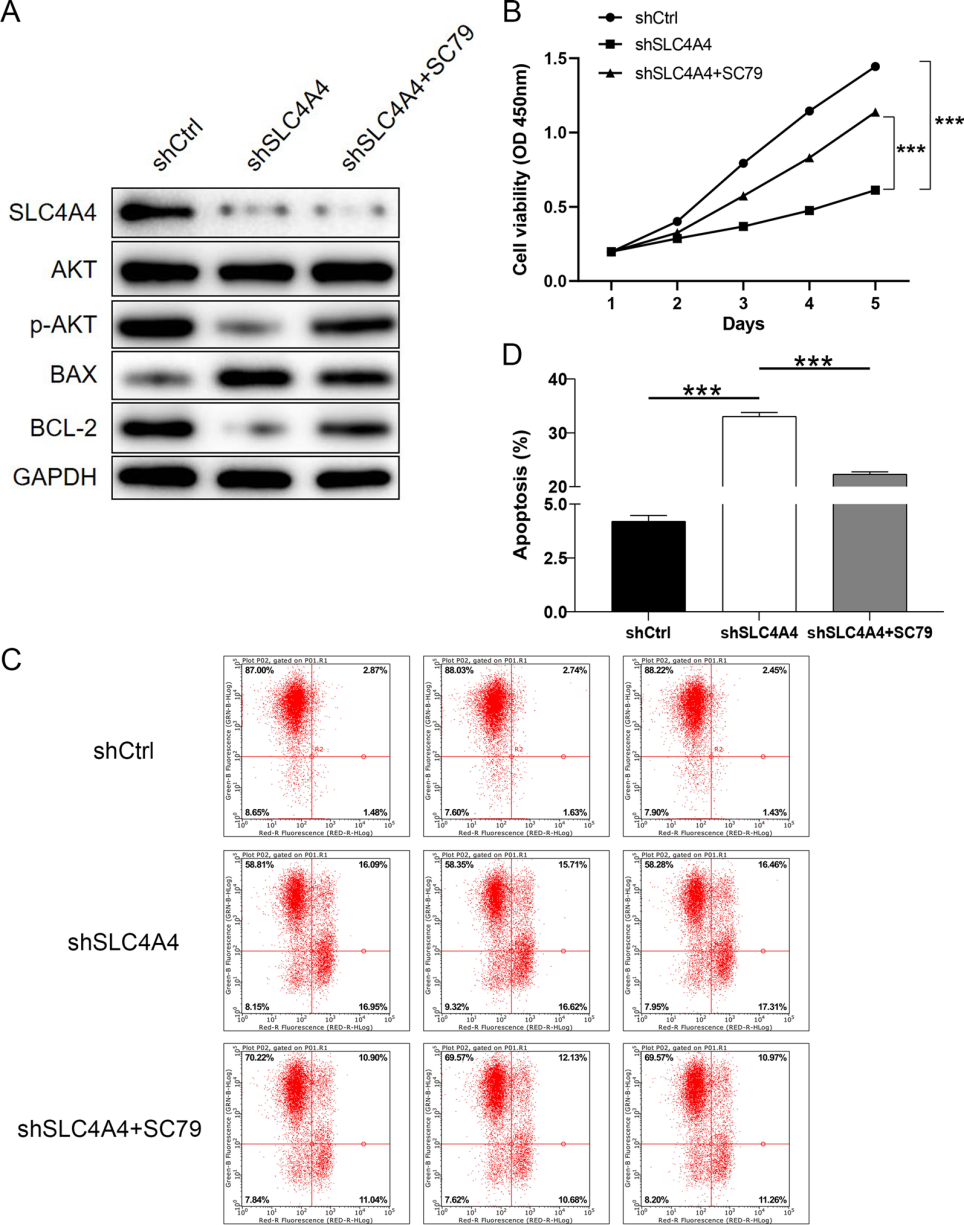
The role of AKT pathway in SLC4A4-mediated PCa progression. **A** Compared to no treatment with SC79, treatment with SC79 of the shSLC4A4 + SC79 group clearly produced the opposite effects on the levels of these proteins. In the presence of SC79, inhibition of SLC4A4 knockdown in DU 145 cells was distinctly reversed, the cell proliferation (**B**) was clearly elevated, and the apoptosis rate (**C-D**) was significantly decreased. Error bars indicate the mean ± SD of at least three replicate experiments. ***P < 0.001. SC79, AKT activator

**Fig. 8:**
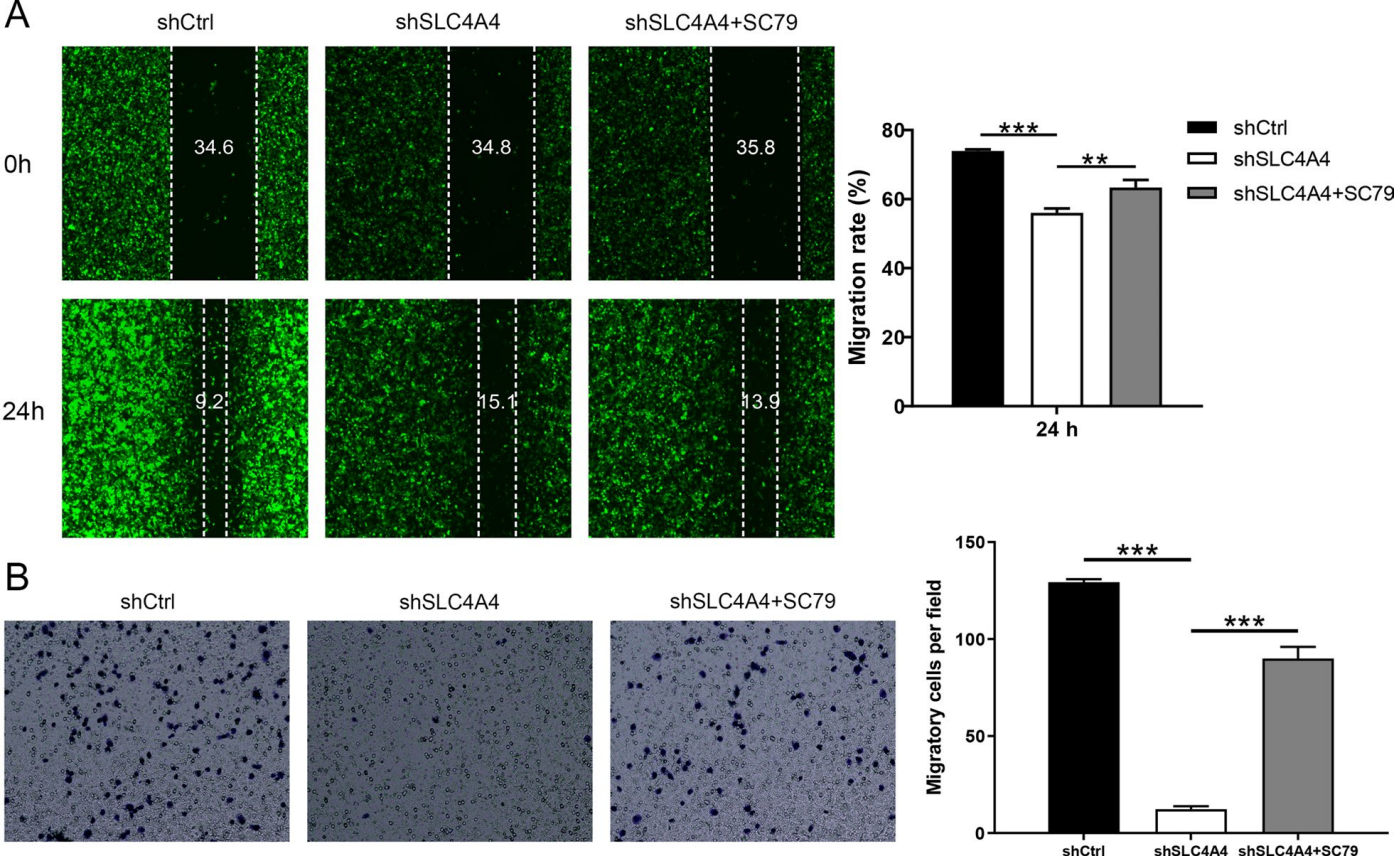
The role of AKT pathway in SLC4A4-mediated PCa progression. In the presence of SC79, inhibition of SLC4A4 knockdown in DU 145 cells was distinctly reversed. **A** Scratch test was conducted to detect the migration abilities of cells treated with SC79. The wounds were photographed at a magnification of 100 × . **B** Transwell assay was employed to evaluate the invasion abilities of cells treated with SC79 at a magnification of 200 × . Error bars indicate the mean ± SD of at least three replicate experiments. ***P < 0.001, **P < 0.01

Activation of PI3K/AKT can lead to diverse biological activities, like immunity, inflammation, cell proliferation, apoptosis, and tumorigenesis [22–24]. In the present study, we detected whether the AKT activator SC79 could reverse the influence of SLC4A4 knockdown on PCa cells. Compared with shCtrl group, the level of SLC4A4 protein was down-regulated, AKT had no significant change, p-AKT expression was down-regulated, BAX expression was up-regulated and BCL-2 expression was down-regulated in shSLC4A4 group (Fig. 7A). Compared to the shSLC4A4 group without SC79 treatment, the treatment with SC79 of the shSLC4A4 + SC79 group produced the opposite effects on the expression of the proteins (p-AKT, BAX, BCL-2). The p-AKT level was clearly enhanced upon SC79 treatment (Fig. 7A). These results demonstrated that SC79 partially reversed the inhibitory action of SLC4A4 knockdown on PCa cells. In order to research the roles of AKT pathway in SLC4A4-mediated cell viability, apoptosis and mobility, rescue experiments were conducted as well. In the existence of SC79, compared with shSLC4A4 group, the cell proliferation was clearly elevated and the apoptosis rate was obviously reduced in shSLC4A4 + SC79 group (Fig. 7B–D). Simultaneously, the migration and invasion abilities of the cells upon the SC79 treatment were meaningfully increased (Fig. 8A, B). Altogether, these findings confirmed that SLC4A4 could promote PCa progression through regulating the AKT pathway.

## Discussion

The genesis and progression of PCa is a complex process containing multiple steps and genes [25, 26], it is therefore of great theoretical and practical significance to illustrate the abnormal expression of genes during prostate carcinogenesis. Our current study verified that SLC4A4 expression was dramatically higher in PCa clinical tissues and cell lines than normal prostate tissues and cells, and increased along with the malignant degree of the tumor. Furthermore, we constructed an SLC4A4 knockdown cell model by lentiviral infection and confirmed the effects of SLC4A4 knockdown on biological behaviours such as proliferation, apoptosis, migration and invasion of PCa cells by Celigo cell counting assay, flow cytometry analysis, wound-healing and Transwell assays. The results indicated that SLC4A4 knockdown inhibited cell proliferation, migration and invasion, while promoting apoptosis. In addition, we constructed an in vivo model of xenografts in nude mice and confirmed that the shSLC4A4 group had a significant inhibitory effect on PCa tumour growth in vivo compared to the shCtrl group, which was also supported by the relatively lower bioluminescence intensity levels and Ki-67 expression in the tumours of the shSLC4A4 group. In short, we demonstrated that knockdown of SLC4A4 could inhibit PCa aggressiveness and progression both in vitro and in vivo.

The acidic and hypoxic tumor environment requires intracellular pH regulation to facilitate tumor development. S. J. Gibbons et al. [27] reported that the electrogenic, sodium-coupled bicarbonate cotransporter, isoform 1 (NBCe1), encoded by *SLC4A4* gene, was expressed in the subtype of interstitial cells of Cajal (ICCs) in the mouse gastrointestinal tract. Mouse ICCs was in charge of the production of slow electrical waves. Moreover, SLC4A4 transcripts expressed in human gastrointestinal smooth muscle cells and mouse ICCs are modifiable isomers. Scott K. Parks et al. [28] demonstrated that SLC4A4 was conducive to the HCO_3_^−^ transports [29] and tumor cell phenotypes, exerting an important effect on the growth and metastasis of breast and colon carcinoma.

Gao et al. [16] indicated that ADH1B, CLCA4, GCG, ZG16, and SLC4A4 were the top five down-regulated molecules in colorectal cancer and SLC4A4 expression was negatively correlated with the prognosis of colorectal cancer patients by survival analysis. Prognostic predictive model according to age, tumor stage, and SLC4A4 expression showed effective performance in the prediction of overall survival among colorectal cancer patients at 1, 3, and 5 year. However, SLC4A4 has been little studied in prostate tumors. The present study is the first to complement the molecular characterization and functional effects of SLC4A4 in PCa tumorigenesis and makes it possible to formulate future strategies for these potentially significant drug targets.

Analysis of clinicopathological data indicated that SLC4A4 was a free-standing prognostic factor of PCa that was meaningfully associated with T Infiltrate, lymphatic metastasis and clinical stage. In other words, high expression of SLC4A4 predicted high malignancy in PCa patients. These results confirmed that the growth, migration and invasion of PCa cells were inhibited in vitro and in vivo after knockdown of SLC4A4. Moreover, our study emphasized that SLC4A4 knockdown induced apoptosis in PCa cells and raised the BAX/BCL-2 ratio, suggesting that SLC4A4 may have an inhibitory effect on apoptosis. Furthermore, the regulatory functions of SLC4A4 in PCa cells were elucidated by a series of gain-of-function analyses.

AKT is an effector molecule of phosphoinositide 3-kinase (PI3K) in the PI3K/AKT/mTOR signalling pathway [30]. Elevated AKT kinase activity in approximately 40% of patients with breast, prostate and gastric cancers has been reported [31]. The AKT pathway serves as an effective medium of signalling from multiple upstream regulatory proteins (e.g. PTEN, PI3K and receptor tyrosine kinases) to some downstream effectors such as GSK3β, FOXO and MDM2, and these signalling pathways can intersect with various other surrogate signalling pathways. Genetic and epigenetic transformations in genes involved in the AKT pathway have been demonstrated to activate AKT in cancer [32], and many lncRNAs can contribute to the over-activation of the AKT signalling pathway through different mechanisms [33, 34]. PI3K/AKT/mTOR signalling pathway is one of the vital intracellular signalling pathways that exert a potent effect on essential cellular functions [35]. Activation of the PI3K/AKT signalling pathway has also been reported as an important cancer-promoting pathway that facilitates cell proliferation and blocks cellular apoptosis [36, 37]. Our current study is consistent with the findings as mentioned above, which all confirm that SLC4A4 could accelerate PCa progression through regulating the AKT pathway.

Some limitations of this study exist, such as the insufficient number of clinical specimens. Besides, prognostic implications of SLC4A4 in PCa and effects of SLC4A4 on different PCa cell lines need to be further investigated. The SLC4A4/NBCe1 has five multiple splice variants, in which expression of the B splice variant in mouse kidney cortical proximal tubule has been presented [38, 39]. Despite the need to better understand PCa progression, the functional mechanisms of SLC4A4 in alternative splicing remain largely elusive [40–42]. The specific downstream genes and regulating mechanisms should be investigated and validated in the future.

## Conclusion

In conclusion, the present study makes the first attempt to link SLC4A4 to human PCa progression. SLC4A4 could be an oncogene to predict tumour malignancy and survival in PCa patients. All these results of this study identified that SLC4A4 knockdown inhibited the occurrence and progression in PCa. SLC4A4 acts as a tumor promotor that accelerates tumor growth, inhibits apoptosis and arrests cell cycle progression among PCa by regulating key elements of the AKT pathway. Thus, SLC4A4 is a promising potential therapeutic target in the treatment of PCa.

## Data Availability

The original data of this article will be made available by the authors, without undue reservations.

## References

[1] Shang Z (2019). LncRNA PCAT1 activates AKT and NF-κB signaling in castration-resistant prostate cancer by regulating the PHLPP/FKBP51/IKKα complex. Nucleic Acids Res.

[2] Rawla P (2019). Epidemiology of prostate cancer. World journal of oncology.

[3] Chen Z (2019). Androgen receptor-activated enhancers simultaneously regulate oncogene TMPRSS2 and lncRNA PRCAT38 in prostate cancer. Cells.

[4] Siegel RL, Miller KD, Jemal A (2016). Cancer statistics, 2016. CA Cancer J Clin.

[5] Stelloo S (2018). Integrative epigenetic taxonomy of primary prostate cancer. Nat Commun.

[6] Abeshouse A, Ahn J, Akbani R, Ally A, Amin S, Andry CD, Annala M, Aprikian A, Armenia J, Arora A, Auman JT (2015). The molecular taxonomy of primary prostate cancer. Cell.

[7] Vishnoi K (2020). Transcription factors in cancer development and therapy. Cancers.

[8] Huynh KW (2018). CryoEM structure of the human SLC4A4 sodium-coupled acid-base transporter NBCe1. Nat Commun.

[9] Zhou J (2020). SLC1A1, SLC16A9, and CNTN3 are potential biomarkers for the occurrence of colorectal cancer. Biomed Res Int.

[10] McIntyre A (2016). Disrupting hypoxia-induced bicarbonate transport acidifies tumor cells and suppresses tumor growth. Can Res.

[11] Xiao W (2019). MiR-223-3p promotes cell proliferation and metastasis by downregulating SLC4A4 in clear cell renal cell carcinoma. Aging.

[12] Yang H (2019). Association of a novel seven-gene expression signature with the disease prognosis in colon cancer patients. Aging.

[13] Gomez-Rueda H (2016). A robust biomarker of differential correlations improves the diagnosis of cytologically indeterminate thyroid cancers. Int J Mol Med.

[14] Gerber JM (2013). Genome-wide comparison of the transcriptomes of highly enriched normal and chronic myeloid leukemia stem and progenitor cell populations. Oncotarget.

[15] Chen X (2020). Prognostic value of SLC4A4 and its correlation with immune infiltration in colon adenocarcinoma. Medical Sci Monit Int Med J Exp Clin Res.

[16] Gao X, Yang J (2020). Identification of genes related to clinicopathological characteristics and prognosis of patients with colorectal cancer. DNA Cell Biol.

[17] Pfaffl MW (2001). A new mathematical model for relative quantification in real-time RT-PCR. Nucl Acids Res.

[18] Pan Y (2014). Sensitive and visible detection of apoptotic cells on Annexin-V modified substrate using aminophenylboronic acid modified gold nanoparticles (APBA-GNPs) labeling. Biosens Bioelectron.

[19] Lim DJ (1993). Growth of an androgen-sensitive human prostate cancer cell line, LNCaP, in nude mice. Prostate.

[20] van Bokhoven A (2003). Molecular characterization of human prostate carcinoma cell lines. Prostate.

[21] Stephenson RA (1992). Metastatic model for human prostate cancer using orthotopic implantation in nude mice. J Natl Cancer Inst.

[22] Porta C, Paglino C, Mosca A (2014). Targeting PI3K/Akt/mTOR signaling in cancer. Front Oncol.

[23] Xu F (2020). Roles of the PI3K/AKT/mTOR signalling pathways in neurodegenerative diseases and tumours. Cell Biosci.

[24] Uko NE (2019). Akt pathway inhibition of the solenopsin analog, 2-dodecylsulfanyl-1,-4,-5,-6-tetrahydropyrimidine. Anticancer Res.

[25] Cucchiara V (2018). Genomic markers in prostate cancer decision making. Eur Urol.

[26] Gandhi J (2018). The molecular biology of prostate cancer: current understanding and clinical implications. Prostate Cancer Prostatic Dis.

[27] Colmenares Aguilar M-G (2021). Expression of the regulated isoform of the electrogenic Na^+^/HCO_3_^-^ cotransporter, NBCe1, is enriched in pacemaker interstitial cells of Cajal. Am J Physiol Gastrointest Liver Physiol.

[28] Parks SK, Pouyssegur J (2015). The Na(+)/HCO3(-) Co-transporter SLC4A4 plays a role in growth and migration of colon and breast cancer cells. J Cell Physiol.

[29] Lee S-K, Boron WF, Parker MD (2013). Substrate specificity of the electrogenic sodium/bicarbonate cotransporter NBCe1-A (SLC4A4, variant A) from humans and rabbits. Am J Physiol Renal Physiol.

[30] Revathidevi S, Munirajan AK (2019). Akt in cancer: mediator and more. Semin Cancer Biol.

[31] Bellacosa A (2005). Activation of AKT kinases in cancer: implications for therapeutic targeting. Adv Cancer Res.

[32] Altomare DA, Testa JR (2005). Perturbations of the AKT signaling pathway in human cancer. Oncogene.

[33] Huang Y (2017). LncRNA AK023391 promotes tumorigenesis and invasion of gastric cancer through activation of the PI3K/Akt signaling pathway. J Exp Clin Cancer Res CR.

[34] Pan W (2019). lncRNA-PDPK2P promotes hepatocellular carcinoma progression through the PDK1/AKT/Caspase 3 pathway. Mol Oncol.

[35] Alzahrani AS (2019). PI3K/Akt/mTOR inhibitors in cancer: at the bench and bedside. Semin Cancer Biol.

[36] Gallardo A (2012). Increased signalling of EGFR and IGF1R, and deregulation of PTEN/PI3K/Akt pathway are related with trastuzumab resistance in HER2 breast carcinomas. Br J Cancer.

[37] Yu X (2020). TSPAN7 exerts anti-tumor effects in bladder cancer through the PTEN/PI3K/AKT pathway. Front Oncol.

[38] Fang L (2018). Expression of the B splice variant of NBCe1 (SLC4A4) in the mouse kidney. Am J Physiol Renal Physiol.

[39] Lee S-K, Boron WF (2018). Exploring the autoinhibitory domain of the electrogenic Na^+^/HCO_3_^-^ transporter NBCe1-B, from residues 28 to 62. J Physiol.

[40] Paschalis A (2018). Alternative splicing in prostate cancer. Nat Rev Clin Oncol.

[41] Zhao J (2020). Systematic profiling of alternative splicing signature reveals prognostic predictor for prostate cancer. Cancer Sci.

[42] Munkley J (2019). Androgen-regulated transcription of drives alternative splicing patterns in prostate cancer. Elife.

